# Automated telemetry reveals age specific differences in flight duration and speed are driven by wind conditions in a migratory songbird

**DOI:** 10.1186/s40462-015-0046-5

**Published:** 2015-08-15

**Authors:** Greg W Mitchell, Bradley K Woodworth, Philip D Taylor, D Ryan Norris

**Affiliations:** Department of Integrative Biology, University of Guelph, Guelph, ON N1G 2W1 Canada; Wildlife Research Division, National Wildlife Research Center, Environment Canada, Ottawa, ON K1H 0H3 Canada; Department of Biology, Acadia University, Wolfville, NS B4P 2R6 Canada; Bird Studies Canada, Port Rowan, ON N0E 1M0 Canada

**Keywords:** Altitude, Aeroecology, Airspeed, Automated telemetry, Crosswinds, Flight costs, Groundspeed, Migration, Songbirds, Tailwinds, Wind support

## Abstract

**Background:**

Given that winds encountered on migration could theoretically double or half the energy expenditure of aerial migrants, there should be strong selection on behaviour in relation to wind conditions aloft. However, evidence suggests that juvenile songbirds are less choosy about wind conditions at departure relative to adults, potentially increasing energy expenditure during flight. To date, there has yet to be a direct comparison of flight efficiency between free-living adult and juvenile songbirds during migration in relation to wind conditions aloft, likely because of the challenges of following known aged individual songbirds during flight. We used an automated digital telemetry array to compare the flight efficiency of adult and juvenile Savannah sparrows (*Passerculus sandwichensis*) as they flew nearly 100 km during two successive stages of their fall migration; a departure flight from their breeding grounds out over the ocean and then a migratory flight along a coast. Using a multilevel path modelling framework, we evaluated the effects of age, flight stage, tailwind component, and crosswind component on flight duration and groundspeed.

**Results:**

We found that juveniles departed under wind conditions that were less supportive relative to adults and that this resulted in juveniles taking 1.4 times longer to complete the same flight trajectories as adults. We did not find an effect of age on flight duration or groundspeed after controlling for wind conditions aloft, suggesting that both age groups were flying at similar airspeeds. We also found that groundspeeds were 1.7 times faster along the coast than over the ocean given more favourable tailwinds along the coast and because birds appeared to be climbing in altitude over the ocean, diverting some energy from horizontal to vertical movement.

**Conclusions:**

Our results provide the first evidence that adult songbirds have considerably more efficient migratory flights than juveniles, and that this efficiency is driven by the selection of more supportive tailwind conditions aloft. We suggest that the tendency for juveniles to be less choosy about wind conditions at departure relative to adults could be adaptive if the benefits of having a more flexible departure schedule exceed the time and energy savings realized during flight with more supportive winds.

**Electronic supplementary material:**

The online version of this article (doi:10.1186/s40462-015-0046-5) contains supplementary material, which is available to authorized users.

## Background

Each year, billions of migrating birds, bats, and insects encounter winds during flight that are on the same order of magnitude as their airspeeds, creating enormous challenges and opportunities that could hypothetically double or half their energy expenditure during flight [1–5]. This observation has resulted in winds figuring prominently in optimal migration theory given their potential effects on flight duration, range, and speed, stopover duration, and orientation (e.g., [3, 6–10]). Moreover, empirical evidence now suggests that winds experienced during migration can be the single largest determinant of annual adult apparent survival in songbirds and can result in carry-over effects between migration and breeding by affecting timing of arrival to the breeding grounds and subsequent breeding productivity [11]. Thus, winds represent a major selective force in migratory behaviour [11–14].

For juvenile songbirds departing on their first autumn migration, decisions regarding timing, flight direction, and flight duration are controlled by an innate genetic program which is later modified by experience [15, 16]. Given the importance of wind for efficient flight, it follows that selection should act strongly on migratory departure decisions for juveniles in relation to winds aloft (e.g., [13, 14]). It also follows that adults and juveniles might have similar departure rules in relation to winds, as both age groups would accrue similar benefits from supportive winds in terms of flight efficiency. However, Mitchell *et al.* [17] found that juvenile Savannah sparrows (*Passerculus sandwichensis*) were much less choosy about wind conditions aloft relative to adults during migratory departure from the breeding grounds. Although this suggests that juveniles were not always departing with supportive winds, there is no direct evidence that this translated to slower migration speeds or longer flight durations during migration. Here, we build upon Mitchell *et al.* [17] by quantifying the costs and benefits in terms of flight efficiency associated with different departure decisions made by adults and juveniles during autumn migration.

The degree to which winds are supportive or unsupportive depends on an individual’s flight trajectory. Therefore, understanding the costs and benefits of departure decisions in relation to winds aloft with respect to individual species and age groups within species is enhanced by the ability to track individuals through space. For larger migratory birds, such as raptors, Global Positioning Satellite (GPS) tracking technology has made it possible to evaluate differences in flight behaviour in relation to wind with respect to age groups [18]. However, given technological constraints on the size of GPS tags [19], studies involving smaller species (<40 g) have largely been restricted to those focusing on departure decisions in relation to winds (e.g., [20–24], although see [25]), and not the wind-mediated costs or benefits of these decisions. Fortunately, the recent development of ground-based automated digital telemetry arrays to track migratory songbirds over broad geographic extents during migration (e.g., [26, 27]), as well as the development of track annotation services to extract modelled wind data for almost any location on earth at multiple altitudes (e.g., [28, 29]), provides new opportunities to evaluate the costs and benefits of wind conditions experienced aloft for small migratory songbirds.

We used an automated digital telemetry array to track the initial stages of autumn migration for adult and juvenile Savannah sparrows, a small (~20 g) grassland songbird. Individuals were tracked nearly 100 km as they completed two successive stages of flight; a 36 ± 2 km flight across the ocean as they travelled from their island breeding grounds to the coast (hereafter, ocean stage) and then a 61 ± 4 km flight as they headed south along the coast (hereafter, coastal stage). Our objectives were two-fold. First, we wanted to establish and demonstrate a simple method to determine the best altitudes from which to measure wind conditions in relation to flight duration and groundspeed for each flight stage. We expected average migratory altitude to be lower for the initial flight over the ocean because birds may be climbing in altitude in search of supportive winds [30–32], whereas, along the coast birds have likely reached their cruising altitude. Second, using a multilevel path modelling framework, we wanted to examine the direct and indirect effects of age, flight stage, and wind conditions aloft on flight duration and groundspeed. For this objective, we hypothesized that juveniles should have longer flight durations and reduced groundspeeds relative to adults because they are more likely to depart under headwind conditions [17]. We also hypothesized that after controlling for the effects of wind, juveniles will have higher groundspeeds because they may be trying to maximize their flight range for a given fuel load [33]. Last, we hypothesized that birds should have longer flight durations and slower groundspeeds over the ocean as opposed to along the coast, because as described above, birds may be climbing in altitude immediately after departure in search of an altitude with optimal winds, slowing their horizontal rate of movement.

## Methods

### Study site, species, and radio transmitters

**Fig. 1:**
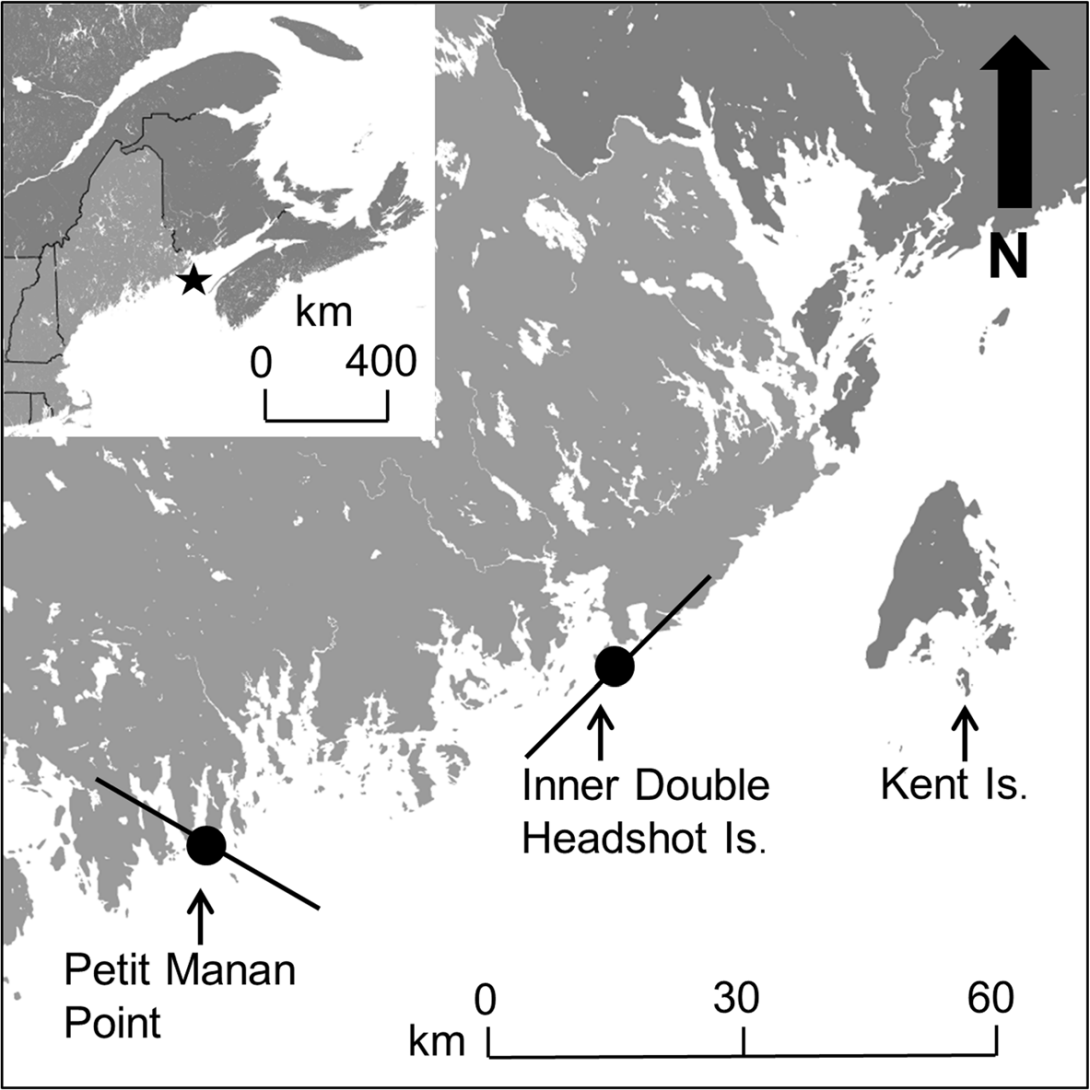
Map of study area. White space represents water and light and dark grey areas represent terrestrial land cover in the USA and Canada, respectively. Solid black circles indicate the locations of automated receiving stations along the coast and solid black lines represent Yagi antenna orientations at each station and the estimated horizontal detection distance from the tower (i.e., 15 km; see Additional file 1: *Estimate of detection range*). Inset represents map of north-eastern USA and Canada. The solid black lines indicate province and state boundaries. The black star indicates the location of the study area presented in the larger map. The black arrow in the top right corner of the larger map represents the direction of geographic north

We studied an island breeding population of Savannah sparrows on Kent Is., New Brunswick, Canada (44°35′ N, 66°45′ W; Fig. 1). The Savannah sparrow is a small (~20 g) grassland songbird that breeds across the northern U.S. and Canada and overwinters in the southern U.S. and Mexico [34].

To track Savannah sparrows during their migratory departure from the breeding grounds we fitted 26 adults and 20 juveniles with 0.62 g digitally-coded radio transmitters (Model NTQB-3-2, Lotek Wireless, Newmarket Ontario) using a figure-eight leg loop harness made of nylon elastic thread (total weight of transmitter + harness = 0.7 g). In a previous study, we found that transmitters with the same mass attached with the same leg loop harness described above, had no effect on multiple measures of pre-migratory body condition in this species [35]. Transmitters were deployed between 25 Aug and 29 Sep 2010. All transmitters were assigned to one of three VHF radio frequencies. Mean body mass (± sd) for males and females fitted with radio transmitters was 20.1 ± 1.2 g and 18.6 ± 1.1 g, respectively. Mean body mass for juveniles was 18.5 ± 1.5 g.

### Automated digital telemetry array

To measure flight durations and groundspeeds we used an automated digital telemetry array comprising three stations on Kent Is. and two along the coast of Maine, USA (Fig. 1 and Additional file 1: Figure S1). Stations on Kent Is. were used to determine the exact time (UTC) of migratory departure, whereas stations on the coast were used to measure the time of arrival at the coast and the duration of travel down the coast. Locations of automated receiving stations along the coast were chosen based on the strong westerly orientation (circular median = 268.2°, rho = 0.90) of 18 adults and 24 juveniles that were tracked during migratory departure from Kent Is. in 2009 using the same transmitters and harness design described above (Additional file 1: *Automated detection of vanishing bearings* and Figures S1-S2). We placed the first coastal receiving station on Inner Double Headshot Is. (44°36′ N, 67°16′ W) and the second station at Petit Manan Point (44°24′ N, 67°54′ W; Fig. 1).

Each station on Kent Is. consisted of four 4-element Yagi antennas positioned near the top of an 8 m high mast. The four antennas were connected to a single automated digital telemetry receiver (Model SRX-600, Lotek Wireless, Newmarket Ontario). Each radio frequency was monitored for 21.6 s continuously every 43.2 s. Further details on the antenna setup and scan cycle for the automated stations on Kent Is. can be found in Mitchell *et al.* [17] and in the (Additional file 1: *Automated detection of vanishing bearings* and Figure S1). Each coastal station consisted of two 9-element Yagi antennas positioned on the top of an 8 m high mast also connected to a single automated digital telemetry receiver(Model SRX-600, Lotek Wireless, Newmarket Ontario). At Inner Double Headshot Is., antennas were oriented parallel to the coast (55° and 235°), and at Petit Manan Point, antennas were oriented perpendicular to the coast (150° and 330°; Fig. 1). At the coastal stations, each radio frequency was monitored for 14 s continuously every 28 s. The approximate horizontal detection range of the 9-element antennas was estimated to be 15 km ([26], Additional file 1: *Estimation of detection distance* and Figure S3).

We defined departure times (UTC) from Kent Is. as the point of maximum signal strength on a characteristic signal strength detection curve for a migratory departure flight [17]. Arrival times (UTC) at Inner Double Headshot Is. and Petit Manan Point were defined by the time of maximum signal strength detection. Flight duration (minutes) was defined as the amount of time it took a bird to fly between two stations. We filtered false-positive signals from our coastal detections by examining each subset of detections individually and ensuring that the time between recorded detections was a multiple of the detected transmitter’s pulse rate. For birds detected at Inner Double Headshot Is., we assumed track orientations of 269°, 275°, and 281° from Kent Is. (relative to geographic north; expected maximum bearing error = 14°) depending on whether a bird was detected by only the southwest directed antenna, both antennas, or only the northeast directed antenna, respectively. An orientation of 275° corresponded to a flight track directly over the station (*n* = 2, 40.5 km flight), while orientations of 269° and 281° corresponded to crossing locations located 7.5 km southwest (*n* = 1, 46.1 km flight) and northeast (*n* = 25, 35.3 km flight) of the station, respectively (Fig. 1). We used the same logic to classify crossing locations for the Petit Manan Point station and assumed the origin was the point of crossing estimated for the Inner Double Headshot Is. station. This resulted in six track orientations: 239° (*n* = 3, 63.8 km flight), 246° (*n* = 5, 62.5 km flight), 247° (*n* = 1, 55.4 km flight), 248° (*n* = 1, 48.8 km flight), 253° (*n* = 9, 62.5 km flight), and 255° (*n* = 1, 55.6 km flight; expected maximum bearing error = 10°). Groundspeeds (m/s) for the ocean and coastal route were defined by distances associated with each of the track orientations listed above divided by flight duration.

### Wind data

To assess the effects of winds aloft on flight duration and groundspeed, we calculated the tailwind and crosswind components experienced during both flight stages. Tailwind components (m/s) were derived using the formula V_w_*cos(β), where V_w_ is wind speed (m/s) and β is the difference between track and wind directions (Additional file 1: *Wind triangles* and Figure S4). Tailwind values ranged from negative to positive with negative values representing the reduction in groundspeed for a given track caused by headwinds, and positive values representing the increase in groundspeed caused by tailwinds. Crosswind components (m/s) were derived using the formula V_w_*sin(β) and represent the strength of the wind component that is blowing perpendicular to a given movement track (Additional file 1: *Wind triangles* and Figure S4).

We obtained wind speed and direction data for the estimated spatial and temporal midpoint of a bird’s track for both the ocean and coastal flight stages from the NCEP/National Oceanic and Atmospheric Administration (NOAA) North American Regional Reanalysis dataset, which has a 32 km spatial resolution and 3 h temporal resolution. The NCEP/NOAA dataset was accessed through the Environmental-Data Automated Track Annotation Service provided by Movebank (www.movebank.org; [29]). Following the methods of Safi *et al.* [36] and Dodge *et al.* [37], all wind conditions were interpolated over space and time (UTC) using inverse distance weighting. We extracted wind speed and direction from altitudes of 10 m and 30 m, as well as wind speed, direction, and geopotential height from 15 pressure levels spanning 1000 mbar to 750 mbar at 25 mbar intervals. Twenty five mbars represents the default resolution for pressure levels available through the track annotation service. We chose a minimum pressure level (maximum altitude) of 750 mbar (~2500 m) because this appears to be the upper limit for passerine migration in North America, particularly during autumn migration [30, 38]. We estimated the average altitude of the winds from each pressure level by taking the average of the geopotential height across all departure evenings. For simplicity and to facilitate communication of methods and results, the different altitudes and pressure levels described above are all hereafter referred to as ‘altitudes’. The estimated spatial and temporal midpoint for each bird’s track are available on Movebank (www.movebank.org, Savannah sparrow, Kent Island, New Brunswick) and are published in the Movebank Data Repository with DOI 10.5441/001/1.82652t83 [39].

### Statistical analysis

**Fig. 2:**
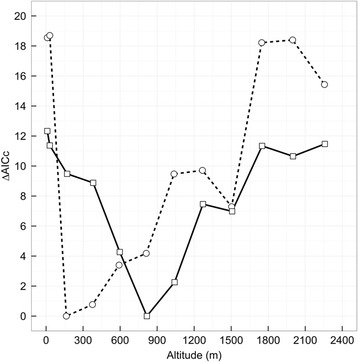
Relationship between ∆AICc values for models relating tail and crosswind components and their interaction to flight times over the ocean (open circles connected by hatched line) and along the coast (open squares connected by solid line) at different altitudes (m) for Savannah sparrows. Wind data is from the NCEP/NOAA dataset and was accessed through the Environmental-Data Automated Track Annotation Service provided by Movebank (32 x 32 km spatial and 3 h temporal resolution; Dodge *et al.* [29]). All wind conditions were interpolated over space and time using inverse distance weighting to the estimated spatial and temporal midpoint of a bird’s track over the ocean and along the coast

All statistical modelling was done in R 3.1.2 [40]. We visually assessed the fit of all models using residual plots. To determine the altitude at which winds were most strongly correlated with flight duration for each flight stage, we modelled flight duration as a function of tailwind component, crosswind component, and their interaction (e.g., [36]) for each of the altitudes described above. For the ocean stage, we included a random effect for ‘nest ID’ to account for potential correlations in flight duration among related individuals (ocean stage: *n* = 8 parent-offspring pairs; lme4 package). We did not include a random effect for ‘nest ID’ for the coastal stage models because we only tracked three parent offspring pairs. Prior to model fitting, we visually assessed the linearity of the relationship between flight duration and the wind components, and included a 2^nd^ order term in the model when there was evidence for a curvilinear relationship. To determine the most parsimonious model for each altitude for both the coastal and ocean flight stages, we carried out an AICc model selection procedure (e.g., [41, 42]), where we compared AICc statistics for all possible model subsets (MuMIn package). In all cases the best fitting model was at least two ΔAICc units less than the null model. After identifying the best model for each altitude, we then compared models among altitudes within each flight stage to determine the altitude for which model fit was best as evidenced by the lowest AICc value (Fig. 2 and Additional file 1: Tables S1 and S2). We then used the wind data from these altitudes (i.e., one altitude per flight stage) to parameterize our multilevel path model.

To examine the direct and indirect effects of age, flight stage, and wind conditions aloft on flight duration and groundspeed we used a multilevel path modelling framework [43]. We analysed flight duration because time is an important currency in optimal migration theory [3, 4]. We analysed groundspeed to test the hypothesis that juveniles may have higher airspeeds relative to adults after controlling for wind conditions aloft, if they are trying to maximize their flight range for a given fuel load [33]. Our analysis of groundspeeds also provides a mechanistic understanding of potential differences in flight durations (or lack thereof) across flight stages.

In both path models we included a random effect for ‘nest ID’ to account for potential correlations in flight duration and groundspeed among related individuals (*n* = 8 parent-offspring pairs) as well as a random effect for ‘individual ID’ to account for repeated measures across flight stages (*n* = 19 individuals). All mixed effects models were fit using the lme4 package. To derive the most parsimonious path model we used an AICc model selection procedure [41, 42, 44]. We started by fitting two global models (one for flight duration and one for groundspeed), both of which included direct effects for flight stage and age on tail and crosswind components, direct effects for flight stage, age, and tail and crosswind components on flight duration and groundspeed, as well as interactions between the tail and crosswind components (e.g., [36]) and between tailwind component and flight stage. Removal of either interaction did not increase the AICc more than two for either model; therefore to simplify our final model selection procedure, we removed these interaction terms from our final analyses. We removed terms associated with uninformative parameter estimates for the wind components first, followed by those with uninformative parameter estimates for flight duration and groundspeed. Terms were removed from the path model if their deletion did not increase the AICc by at least two. We did not model average because the top models were all nested versions of the preceding models [45]. Parameter estimates presented in the results are for standardized data. All mean values are reported with ± 1 standard deviation and all median values are presented with ranges in parentheses.

## Results

### Flight trajectories

We tracked nine juveniles and 19 adults flying west-northwest from Kent Is. to the coast (*n* = 28, mean distance = 36.6 ± 2.4 km, mean track direction = 280 ± 3°) and then tracked five of these juveniles and 14 of these adults as they continued their migration southwest along the coast (*n* = 19, mean distance = 61.2 ± 3.8 km, mean track direction *n* = 249 ± 5°). We were unable to track flights along the coast for the remaining four juveniles and five adults that we originally tracked from Kent Is. to the coast because they were not detected by the southernmost receiving station on the coast. For the 19 birds that we did track flying down the coast, mean track length and flight duration from Kent Is. to the southernmost receiving station was 97.6 ± 10.5 km and 134.1 ± 45.8 min, respectively. Average date of migratory departure for all birds that were detected by a coastal receiving station was Oct 03 ± 9 days. We also tracked one bird flying between Inner Double Headshot Is. and Petit Manan Point 15 days after it originally departed Kent Is. This bird initially flew by the Inner Double Headshot Is. station at 01:54 UTC. In comparison, eight other birds that were tracked from Kent Is. to the coast on the same evening all flew by the Inner Double Headshot Is. station between 23:41 UTC and 24:17 UTC, suggesting the former bird was likely well into its migratory flight when it was detected. Therefore, we included this bird’s coastal flight in our final analysis (*n* = 20 for the coastal stage) as it had likely reached its cruising altitude when it was detected by the Inner Double Headshot Is. station (see paragraph below).

### Flight altitudes

We found that the nature of the relationship between flight duration and wind and the best altitude from which to measure wind data varied across flight stages. Specifically, for the ocean stage we found that a linear model relating flight duration to tailwind component, where tailwind component was measured at an altitude of 164 ± 44 m (1000 mbar), performed best (Fig. 2 and Additional file: 1: Table S1). With respect to the coastal stage, we found that a linear model that included a curvilinear term for tailwind component, where tailwind component was measured at an altitude of 817 ± 46 m (925 mbar) performed best (Fig. 2 and Additional file 1: Table S2).

### Factors affecting flight duration

**Fig. 3:**
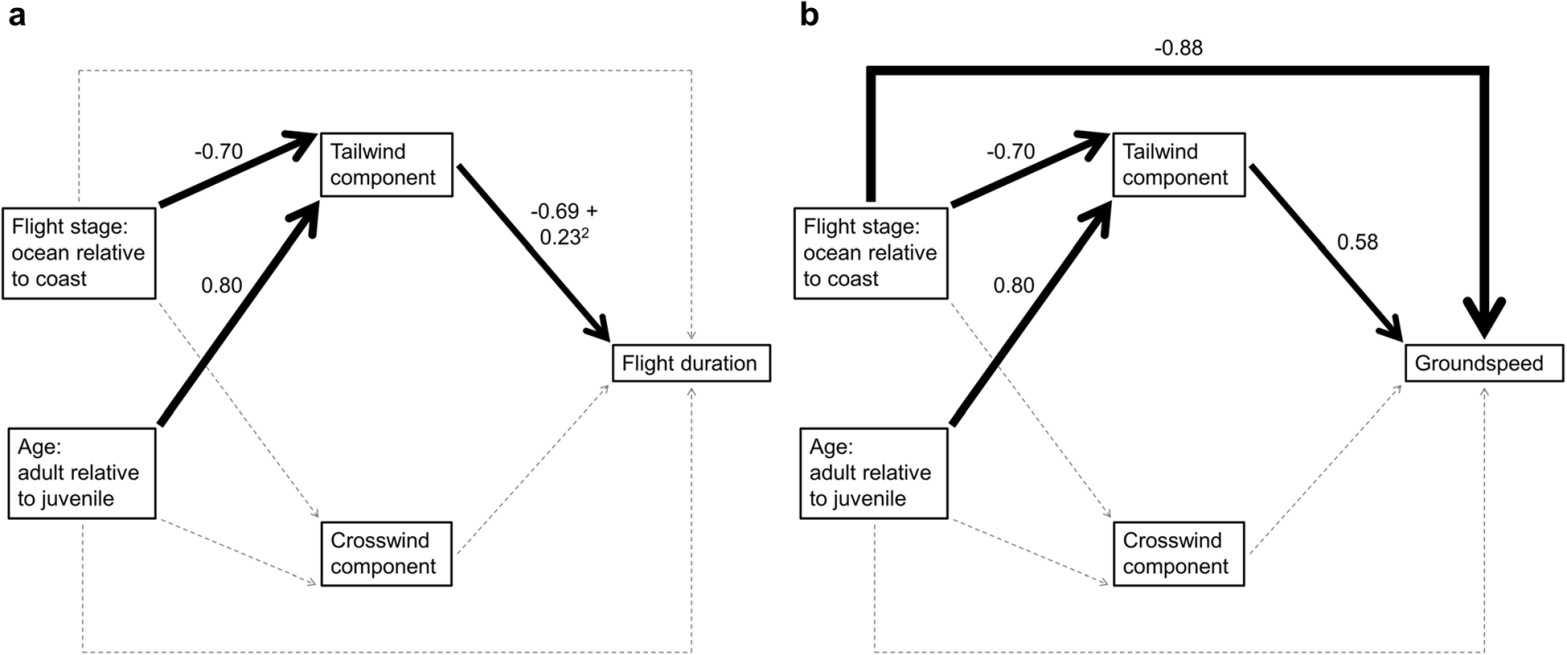
Path diagrams showing factors affecting **a** flight duration and **b** groundspeed. Path models represent the most parsimonious models from our AICc model selection procedure (Tables 1 and 2). Light grey hatched lines represent paths for uninformative parameter estimates and black lines represent paths for informative parameter estimates. For the latter, each line is scaled in width relative to the standardized path coefficient located above each line

**Fig. 4:**
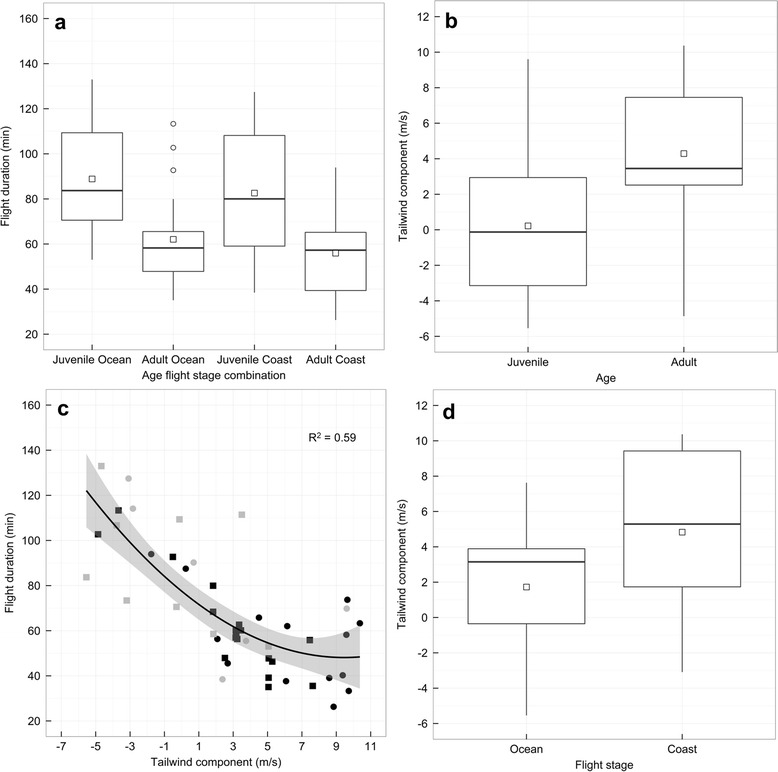
**a** Box plot illustrating differences in flight durations between adults and juveniles for flights over the ocean and along the coast. The hollow squares and horizontal lines within each box represent the mean and median of the variable of interest, respectively. Hollow circles represent values lying outside 1.5 * the interquartile range. **b** Box plot illustrating difference in tailwind components experienced by juvenile and adult birds pooled across routes. **c** Scatter plot with curvilinear regression line and 95 % confidence interval illustrating relationship between flight duration and tailwind component. Squares and circles represent juvenile and adult birds, respectively, while grey and black points represent ocean and coastal routes, respectively. R^2^ represents the marginal deviance explained. **d** Box plot illustrating difference in tailwind components experienced by birds over the ocean and along the coast

We found that both age and flight stage influenced flight duration, but that these effects were indirectly mediated by the tailwind components experienced aloft (Fig. 3a and Table 1). The net effect of this indirect relationship was that juveniles, on average, took 25 min longer to fly between Kent Is. and the coast and 23 min longer to move down the coast relative to adults (median flight duration: juveniles ocean = 90 min (53–113 min); adults ocean = 58 min (35–113 min); juveniles coast = 80 min (39–128 min); adults coast = 56 min (26–94 min); Fig. 4a). Specifically, we found that juveniles tended to depart with less supportive tailwind components relative to adults (intercept = −0.13; β_age:adult_ = 0.80; Fig. 4b and Table 1), which strongly increased their flight durations (intercept = −0.23; β_tailwind_ = −0.69; β_tailwind_^2^ = 0.23; Fig. 4c and Table 1). We also found that birds flying over the ocean tended to experience less supportive tailwind components than along the coast (β_stage:ocean_ = −0.70; Fig. 4d and Table 1). To examine if the flight stage effect was confounded by the use of winds from two different altitudes, we refit the model examining the effects of flight stage on the tail and crosswind components using only winds from either 1000 mbar or 925 mbar. In each model, the flight stage effect was still present and effect sizes were similar (Additional file 1: Table S3).

**Table 1:** AICc model selection results for path analytic models examining factors affecting flight duration across the ocean and along the coast

Path model equations	K	AICc	ΔAICc	W	Cumulative W	Fisher’s C
tailwind ~ stage + age	16	71.64	0	0.56	0.56	22.09
flight duration ~ tailwind + tailwind^2^						
tailwind ~ stage + age	17	72.37	0.73	0.39	0.95	17.97
flight duration ~ age + tailwind + tailwind^2^						
tailwind ~ stage + age	18	76.69	5.05	0.04	0.99	17.11
flight duration ~ stage + age + tailwind + tailwind^2^						
tailwind ~ stage + age	19	79.00	7.36	0.01	1	13.86
flight duration ~ stage + age + tailwind + tailwind^2^ + crosswind						
tailwind ~ stage + age	20	83.79	12.15	0	1	12.68
crosswind ~ age						
flight duration ~ stage + age + tailwind + tailwind^2^ + crosswind						
tailwind ~ stage + age	21	86.73	15.09	0	1	9.19
crosswind ~ stage + age						
flight duration ~ stage + age + tailwind + tailwind^2^ + crosswind						
tailwind ~ stage + age	14	120.04	48.40	0	1	79.31

k represents the number of parameters in each path model. W and Cumulative W represent Akaike weights and cumulative model weights, respectively. Fisher’s C statistic = −2*ln(model likelihood). The null model is defined by the top model with paths to flight duration removed. ^2^ indicates curvilinear parameter coefficient

### Factors affecting groundspeed

**Fig. 5:**
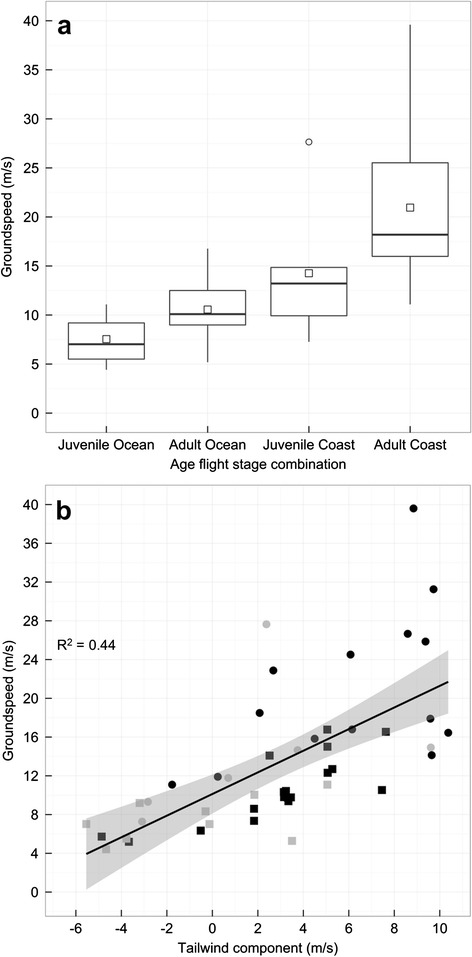
**a** Box plot illustrating differences in groundspeeds between adults and juveniles for flights over the ocean and along the coast. The hollow squares and horizontal lines within each box represent the mean and median groundspeed, respectively. Hollow circles represent values lying outside 1.5 * the interquartile range. **b** Scatter plot and regression line with 95 % confidence interval illustrating relationship between groundspeed and tailwind component. Squares and circles represent adult and juvenile birds, respectively, while grey and black points represent ocean and coastal routes, respectively. R^2^ represents the marginal deviance explained from the partial regression where the effect of route has been removed from both groundspeed and tailwind component

Similar to flight duration, we found that both age and flight stage influenced groundspeed, and that these effects were indirectly mediated by the tailwind components experienced aloft (Fig. 3b and Table 2). In fact, given the model structure, the effects of age and flight stage on the tailwind component experienced during flight are identical to those reported for the flight duration model above (Tables 1 and 2). The net effect of these indirect relationships was that adults flew 3 m/s and 5 m/s faster on average than juveniles over the ocean and down the coast, respectively (median groundspeed: juvenile ocean = 7 m/s (4–11 m/s); adult ocean = 10 m/s (5–16 m/s); juvenile coast = 13 m/s (7–28 m/s); adult coast = 18 m/s (11–40 m/s); Fig. 5a). Specifically, and again similar to our model involving flight duration, juveniles tended to depart and fly with less supportive tailwind components relative to adults which had the effect of decreasing their groundspeeds relative to adults (intercept = 0.51; β_tailwind_ = 0.58; Fig. 5b and Table 2). Different from our flight duration model, we also found a direct effect of flight stage on log groundspeed, where after controlling for the effect of tailwind component experienced aloft, groundspeed was much faster along the coast than over the ocean (β_route:ocean_ = −0.87; Fig. 5a and Table 2). The net effect of the direct and indirect effects of flight stage on groundspeed was that birds were flying 7 m/s faster, on average, down the coast as opposed to over the ocean (median groundspeed: ocean = 10 m/s (4–17 m/s); coast = 17 m/s (7–40 m/s); Fig. 5a).

**Table 2:** AICc model selection results for path analytical models examining factors affecting groundspeed over the ocean and along the coast

Path model equations	K	AICc	ΔAICc	W	Cumulative W	Fisher’s C
tailwind ~ stage + age	16	67.36	0	0.70	0.68	17.81
groundspeed ~ stage + tailwind						
tailwind ~ stage + age	17	69.34	1.98	0.26	0.96	14.94
groundspeed ~ stage + age + tailwind						
tailwind ~ stage + age	18	73.44	6.08	0.03	0.99	13.86
groundspeed ~ stage + age + tailwind + crosswind						
tailwind ~ stage + age	19	75.58	8.22	0.01	1	10.44
groundspeed ~ stage + age + tailwind + crosswind						
crosswind ~ age						
tailwind ~ stage + age	20	80.30	12.94	0	1	9.19
groundspeed ~ stage + age + tailwind + crosswind						
crosswind ~ stage + age						
tailwind ~ stage + age	13	115.39	48.03	0	1	78.69

k represents the number of parameters in each path model. W and Cumulative W represent Akaike weights and cumulative model weights, respectively. Fisher’s C statistic = −2*ln(model likelihood). The null model is defined by the top model with paths to groundspeed removed

## Discussion

Our results provide the first evidence that adult songbirds are considerably more efficient in their migratory flight relative to juveniles, and that this difference is driven by wind conditions experienced aloft. More specifically, we found that juveniles flew with less supportive tailwind components relative to adults, resulting in juveniles taking 1.4 times as long on average to cover the same distances as adults, or alternatively, that adults were travelling 1.4 times faster on average than juveniles along the same flight trajectories. This could translate into important differences in distances flown. For example, if we assume that both age groups departed with similar fuel loads and had similar flight durations (i.e., civil sunrise–civil sunset = 10.5 h on Oct 03), and given that median rates of movement down the coast were 65.5 km/h for adults and 47.6 km/h for juveniles (Fig. 5b), adults on average would have covered an additional 188 km during their first migratory flight relative to juveniles. If this pattern persisted over the course of the entire migration, juveniles would have had to stopover more frequently, potentially increasing their predation risk and total energy expenditure [12, 46–48].

### Why do juveniles depart with less supportive winds?

Given the importance of energy for migration, natural selection is hypothesized to act strongly on migratory behaviour in relation to winds [12–14]. We suggest that the tendency for juveniles to be less choosy about wind conditions at departure relative to adults could be adaptive if the benefits of having a more flexible departure schedule exceed the time and energy savings realized during flight with more supportive winds. For example, juveniles may choose to depart as soon as possible if predation rates are high or perceived as high, or if energy loss associated with cold night time temperatures while waiting for more favourable conditions is large [46, 47]. Alternatively, intraspecific competition for resources on the island breeding grounds could be intense [49, 50], forcing juveniles to depart as soon as they are capable of doing so.

### Implications of results for airspeeds

Theoretical predictions regarding optimal airspeeds (flight speed in still air or effort) in relation to wind suggest that birds should increase their airspeeds as tailwind components become more negative to maximize flight range for a given fuel load [6, 33, 48]. This is because as wind support decreases and headwinds begin to increase in strength, birds must fly faster to maximize the ratio of speed to power. In our study we found that juveniles generally flew with less supportive winds than adults (Fig. 4a), however, after controlling for the effect of tailwind component, we did not find a direct effect of age on groundspeed, which would have indicated potential differences in airspeed. Therefore, our results suggest that juveniles were not attempting to maximize their flight range with respect to their departure fuel loads in relation to wind. The lack of difference in airspeeds among age groups further suggests that differences in flight durations and groundspeed can act as a proxy for energy expenditure across ages. This means that juveniles, on average, were spending 1.4 times more energy to complete the same flight distances as adults.

### Flight altitudes

We found that groundspeeds along the coast were 1.7 times higher on average than those over the ocean. This explains why we did not observe a flight stage effect on flight duration, despite the coastal flight stage being approximately 25 km longer in length. Although we predicted shorter flight durations along the coast, this null result still lends support to our hypothesis for a flight stage effect being driven by differences in climbing behaviour. More importantly, after controlling for the effect of more supportive tailwinds along the coast, our path model revealed a direct effect of flight stage on groundspeed. This observation along with our assessment of the best altitude at which to sample winds for our model of flight duration strongly supports our hypothesis that stage specific differences in groundspeed are driven by differences in climbing behaviour. Specifically, we found that the best altitudes at which to measure winds for the ocean flight were 164 m and 376 m (1000 mbar and 975 mbar, respectively; Fig. 2), while for the coastal stage, the best altitude was 817 m (925 mbar; Fig. 2). If we interpret these results as the average altitude of flight over each stage, then it suggests that birds were climbing in altitude during their ocean flight. Given that small songbirds can climb at a rate of 1–2 m/s [51, 52], the birds in our study could easily have achieved an altitude of approximately 800 m by the time they reach the coast. Therefore, our results support the hypothesis that slower rates of movement over the ocean relative to the coast, after controlling for wind, are because birds are putting energy into both their horizontal and vertical movement during their ocean flight as opposed to only horizontal movement along the coast.

We present a simple method for probing the atmosphere to find the best wind data for a given metric of flight performance, which simultaneously provides insights into average flight altitudes for a given flight track. We want to stress that this is an approximate estimate of average flight altitude. For example, there is hourly, daily, and by extension, weekly changes in optimal flight altitudes in relation to wind conditions experienced aloft as pressure systems move across the surface of the earth, which would cause variability in the selection of flight altitudes [53]. Moreover, the curvilinear relationship we observed between flight duration and tailwind component also suggests some error in our selection of average flight altitudes for at least some birds, as it is very unlikely that birds would slow their groundspeeds in favourable wind conditions (Fig. 1b). Instead, we suggest that these birds may have sought out the first “acceptable” altitude in terms of wind support [31, 32], which was likely below 817 m and had lower tailwind support than the winds at 817 m. If this interpretation is correct, then it further suggests that Savannah sparrows might stop searching for better wind conditions if they find a tailwind component of approximately 6–7 m/s, as this is the point where the relationship between flight duration and tailwind component begins to level off (Fig. 4c).

### Implications of results for radar studies

Radar studies of passerine migration show large amounts of variation in flight speeds in the autumn, both under headwind conditions when the seasonal availability of more favourable tailwinds is low, as well as when tailwinds are more readily available (e.g., [5, 54–56]). While this variability is likely, at least partly, due to differences in migration strategies among species, our results suggest that some of the variation might be caused by differences in migration strategies among age groups within species. Further research is needed to determine if this is a general phenomenon within other songbirds.

### Risk averse flight trajectories

We tracked 73 % of the adults and 45 % of the juveniles that were originally radio tagged flying almost directly west from Kent Is. to the coast. This suggests that this is the preferred migratory track for the population. While we cannot definitively say what trajectory the other 55 % of juveniles had, we suggest it was probably north-northwest based on our observations of approximate vanishing bearings (Additional file 1: Figure S2). We also suggest the juveniles that were never detected again after departure from Kent Is. also likely departed with lower wind support relative to adults based on the results of Mitchell *et al.* [17], making the results of our analysis generalizable across all juvenile birds in our study population. Our observation of a westerly orientation of birds upon departure followed by a southwest orientation along the coast suggests that Savannah sparrows in our study population are risk averse with respect to flights over open water or that potential time savings accrued by flying southwest over open water are smaller than the benefits gained from taking a longer route with a shorter flight distance over water.

## Conclusions

In conclusion, our results provide the first evidence that adult songbirds have considerably more efficient migratory flights than juveniles, and that this efficiency is driven by the selection of more supportive tailwind conditions aloft. For juveniles, being less choosy about tailwind conditions resulted in 1.4 times greater energy expenditure over the same flight distances as adults and likely resulted in an average reduction in flight distance of 188 km relative to adults during the first migratory flight of the season. We also provide a simple method to use readily available atmospheric data (see [26, 27]) to estimate the average flight altitude of passerines. This is directly relevant for other studies modelling flight trajectory data from automated telemetry arrays, but also has important implications for assessing collision risks with towers, buildings, and other tall infrastructure [57]. Given present challenges of tracking small (<40 g) migratory animals for which species, sex, and age are known [19], we suggest automated telemetry arrays provide new opportunities to test multiple hypotheses associated with optimal migration theory (e.g., [33]) and to better understand how winds have shaped migratory behaviour through natural selection. Understanding these factors will ultimately improve our understanding of species, sex, and age-specific impacts of potential climate-driven changes in atmospheric conditions [58, 59].

## Additional file

Additional file 1:
**Supporting information regarding automated detection of vanishing bearings, estimation of detection distance, and wind triangles.** This file also contains the following tables and figures: **Table S1.** AICc model selection results for best fitting linear mixed effects model relating flight time across the ocean to tail and crosswind components from different altitudes; **Table S2.** AICc model selection results for best fitting linear model relating flight time along the coast to tail and crosswind components from different altitudes; **Table S3.** Results for models describing the effects of age and flight stage on the tailwind component experienced aloft; **Figure S1.** Map of Kent Is. depicting the locations and orientation of antennas on each station from 2009; **Figure S2.** Rose diagrams (circular histograms) illustrating vanishing bearings of Savannah sparrows departing Kent Is. in 2009 and 2010; **Figure S3.** Density plot of estimated detection distances for Savannah sparrows departing Kent Is. in 2010; **Figure S4.** Hypothetical wind triangles or vector addition diagrams illustrating a (A) positive and (B) negative tailwind component.

## References

[1] Liechti F (2006). Birds: blowin’ by the wind?. J Ornithol.

[2] Srygley RB, Dudley R (2008). Optimal strategies for insects migrating in the flight boundary layer: mechanisms and consequences. Integr Comp Biol.

[3] Hedenström A (2009). Optimal migration strategies in bats. J Mammal.

[4] Alerstam T (2011). Optimal bird migration revisited. J Ornithol.

[5] Alerstam T, Chapman JW, Bäckman J, Smith AD, Karlsson H, Nilsson C, et al. Convergent patterns of long-distance nocturnal migration in noctuid moths and passerine birds. P Roy Soc Lond B Bio. 2011;278:3074–80. 10.1098/rspb.2011.0058 PMC3158935 21389024

[6] Liechti F, Hedenström A, Alerstam T (1994). Effects of sidewinds on optimal flight speed of birds. J Theor Biol.

[7] Liechti F (1995). Modelling optimal heading and airspeed of migrating birds in relation to energy expenditure and wind influence. J Avian Biol.

[8] Liechti F, Bruderer B (1998). The relevance of wind for optimal migration theory. J Avian Biol.

[9] Weber TP, Alerstam T, Hedenström A (1998). Stopover decisions under wind influence. J Avian Biol.

[10] Weber TP, Hedenström A (2000). Optimal stopover decisions under wind influence: the effects of correlated winds. J Theor Biol.

[11] Drake A, Crock CA, Quinlan SP, Martin M, Green DJ G (2014). Wind speed during migration influences the survival, timing of breeding, and productivity of a Neotropical migrant, Setophagia petechial. PLoS One.

[12] Alerstam T (1979). Wind as selective agent in bird migration. Ornis Scand.

[13] Erni B, Liechti F, Bruderer B (2005). The role of wind in passerine autumn migration between Europe and Africa. Behav Ecol.

[14] McLaren JD, Shamoun-Baranes J, Bouten W (2012). Wind selectivity and partial compensation for wind drift among nocturnally migrating passerines. Behav Ecol.

[15] Berthold P (2001). Bird migration.

[16] Pulido F (2007). The genetics and evolution of avian migration. Bioscience.

[17] Mitchell GW, Newman AE, Wikelski M, Norris DR (2012). Timing of breeding carries over to influence migratory departure in a songbird: an automated radiotracking study. J Anim Ecol.

[18] Thorup K, Alerstam T, Hake M, Kjellén N (2003). Bird orientation: compensation for wind drift in migrating raptors is age dependent. P Roy Soc Lond B Bio.

[19] Bridge ES, Thorup K, Bowlin MS, Chilson PB, Diehl RH, Fléron RW, et al. Technology on the move: recent and forthcoming innovations for tracking migratory birds. Bioscience. 2011;61:689–98.

[20] Åkesson S, Walinder G, Karlsson L, Ehnbom S (2002). Nocturnal migratory flight initiation in reed warblers Acrocephalus scirpaceus: effect of wind on orientation and timing of migration. J Avian Biol.

[21] Schaub M, Liechti F, Jenni L (2004). Departure of migrating European robins, Erithacus rubecula, from a stopover site in relation to wind and rain. Anim Behav.

[22] Smith AD, McWilliams SR (2014). What to do when stopping over: behavioral decisions of a migrating songbird during stopover are dictated by initial change in their body condition and mediated by key environmental conditions. Behav Ecol.

[23] Schmaljohann H, Naef-Daenzer B (2011). Body condition and wind support initiate shift in migratory direction and timing of nocturnal departure in a free flying songbird. J Anim Ecol.

[24] Delingat J, Bairlein F, Hedenström A (2008). Obligatory barrier crossing and adaptive fuel management in migratory birds: the case of the Atlantic crossing in northern wheatears (Oenanthe oenanthe). Behav Ecol Sociobiol.

[25] Karlsson H, Henningsson P, Bäckman J, Hedenström A, Alerstam T (2010). Compensation for wind drift by migrating swifts. Anim Behav.

[26] Taylor PD, Mackenzie SA, Thurber BG, Calvert AM, Mills AM, McGuire LP, et al. Landscape movements of migratory birds and bats reveal an expanded scale of stopover. PloS One. 2011;6(11), e27054. 10.1371/journal.pone.0027054 PMC3207824 22073253

[27] Woodworth BK, Mitchell GW, Norris DR, Francis CM, Taylor PD (2014). Patterns and correlates of songbird movements at an ecological barrier during autumn migration assessed using landscape‐and regional‐scale automated radiotelemetry. Ibis.

[28] Kemp MU, van Loon EE, Shamoun-Baranes J, Bouten W (2011). RNCEP: global weather and climate data at your fingertips. Methods Ecol Evol.

[29] Dodge S, Bohrer G, Weinzierl R, Davidson SC, Kays R, Douglas D, et al. The environmental-data automated track annotation (Env-DATA) system: linking animal tracks with environmental data. Movement Ecology. 2013;1(1):3. 10.1186/2051-3933-1-3 PMC4337772 25709817

[30] Gauthreaux SA (1991). The flight behavior of migrating birds in changing wind fields: radar and visual analyses. Am Zool.

[31] Mateos-Rodríguez M, Liechti F (2012). How do diurnal long-distance migrants select flight altitude in relation to wind?. Behav Ecol.

[32] Kemp MU, Shamoun‐Baranes J, Dokter AM, Loon E, Bouten W (2013). The influence of weather on the flight altitude of nocturnal migrants in mid‐latitudes. Ibis.

[33] Pennycuick CJ (1978). Fifteen testable predictions about bird flight. Oikos.

[34] Wheelwright NT, Rising JD. Savannah sparrow (Passerculus sandwichensis). In: Poole A, editor. The birds of North America online. Ithaca: Cornell Lab of Ornithology; 2008. http://bna.birds.cornell.edu/bna/species/045. Accessed 01 January 2015.

[35] Rae LF, Mitchell GW, Mauck RA, Guglielmo CG, Norris DR (2009). Radio transmitters do not affect the body condition of Savannah sparrows during the fall premigratory period. J Field Ornithol.

[36] Safi K, Kranstauber B, Weinzierl R, Griffin L, Rees E, Cabot D, et al. Flying with the wind: scale dependency of speed and direction measurements in the modelling of wind support in avian flight. Movement Ecology. 2013;1(1):4. 10.1186/2051-3933-1-4 PMC4337751 25709818

[37] Dodge S, Bohrer G, Bildstein K, Davidson SC, Weinzierl R, Bechard MJ, et al. Environmental drivers of variability in the movement ecology of turkey vultures (Cathartes aura) in North and South America. Philos T Roy Soc B. 2014;369:20130195. 10.1098/rstb.2013.0195 PMC3983930 24733950

[38] Williams TC, Williams JM, Ireland LC, Teal JM (1977). Autumnal bird migration over the Western North Atlantic Ocean. Am Birds.

[39] Mitchell GW, Woodworth BK, Taylor PD, Norris DR. Data from: automated telemetry reveals age specific differences in flight duration and speed are driven by wind conditions in a migratory songbird. Movebank data repository. doi:10.5441/001/1.82652t83. 10.1186/s40462-015-0046-5 PMC4537592 26279850

[40] Core Team R (2014). R: a language and environment for statistical computing.

[41] Bartlam‐Brooks HL, Beck PS, Bohrer G, Harris S (2013). In search of greener pastures: using satellite images to predict the effects of environmental change on zebra migration. J Geophys Res-Biogeo.

[42] Bohrer G, Beck PS, Ngene SM, Skidmore AK, Douglas-Hamilton I. Elephant movement closely tracks precipitation-driven vegetation dynamics in a Kenyan forest-savanna landscape. Movement Ecology. 2014; 2(1):2. 10.1186/2051-3933-2-2 PMC4267703 25520813

[43] Shipley B (2009). Confirmatory path analysis in a generalized multilevel context. Ecology.

[44] Shipley B (2013). The AIC, model selection method applied to path analytic models compared using a d-separation test. Ecology.

[45] Arnold TW (2010). Uninformative parameters and model selection using Akaike’s information criterion. J Wildlife Manage.

[46] Wikelski M, Tarlow EM, Raim A, Diehl RH, Larkin RP, Visser GH (2003). Avian metabolism: costs of migration in free-flying songbirds. Nature.

[47] Woodworth BK, Francis CM, Taylor PD (2014). Inland flights of young red‐eyed vireos *Vireo olivaceus* in relation to survival and habitat in a coastal stopover landscape. J Avian Biol.

[48] Hedenström A, Alerstam T (1995). Optimal flight speed of birds. Philos T Roy Soc B.

[49] Rappole JH, Warner DW (1976). Relationships between behavior, physiology and weather in avian transients at a migration stopover site. Oecologia.

[50] Woodrey MS (2000). Age-dependent aspects of stopover biology of passerine migrants. Stud Avian Biol.

[51] Able KP (1977). The flight behaviour of individual passerine nocturnal migrants: a tracking radar study. Anim Behav.

[52] Hedenström A, Alerstam T (1992). Climbing performance of migrating birds as a basis for estimating limits for fuel-carrying capacity and muscle work. J Exp Biol.

[53] Dokter AM, Shamoun-Baranes J, Kemp MU, Tijm S, Holleman I (2013). High altitude bird migration at temperate latitudes: a synoptic perspective on wind assistance. PLoS One.

[54] Bloch R, Bruderer B (1982). The air speed of migrating birds and its relationship to the wind. Behav Ecol Sociobiol.

[55] Larkin RP (1991). Flight speeds observed with radar, a correction: slow “birds” are insects. Behav Ecol Sociobiol.

[56] Karlsson H, Nilsson C, Bäckman J, Alerstam T (2011). Nocturnal passerine migration without tailwind assistance. Ibis.

[57] Calvert AM, Bishop CA, Elliot RD, Krebs EA, Kydd TM, Machtans CS, et al. A synthesis of human-related avian mortality in Canada. Avian Conserv Ecol. 2013;8:11.

[58] Corti S, Molteni F, Palmer TN (1999). Signature of recent climate change in frequencies of natural atmospheric circulation regimes. Nature.

[59] Sousounis PJ, Grover EK (2002). Potential future weather patterns over the Great Lakes region. J Great Lakes Res.

