# Supplementary material for: Automated telemetry reveals age specific differences in flight duration and speed are driven by wind conditions in a migratory songbird
Source: Mov Ecol. 2015 Aug 15;3:19. doi: 10.1186/s40462-015-0046-5 (PMC4537592; doi:10.1186/s40462-015-0046-5)

Additional file

Automated telemetry reveals age specific differences in flight duration and speed are driven by wind conditions in a migratory songbird

Greg W Mitchell[1,2*] (Gregory.Mitchell@ec.gc.ca)

Bradley K Woodworth[1,3] (bwoodwor@uoguelph.ca)

Philip D Taylor[3,4] (Philip.Taylor@acadiau.ca)

D Ryan Norris[1] (rnorris@uoguelph.ca)

[1] *Department of Integrative Biology, University of Guelph, Guelph, Ontario, Canada N1G 2W1*

[2] *Wildlife Research Division, National Wildlife Research Center, Environment Canada, Ottawa, Ontario, Canada, K1H 0H3*

[3] *Department of Biology, Acadia University, Wolfville, Nova Scotia, Canada B4P 2R6*

[4] *Bird Studies Canada, Port Rowan, Ontario, Canada, N0E 1M0*

**Author for correspondence (Gregory.Mitchell@ec.gc.ca).*

Keywords: altitude; aeroecology; airspeed; automated telemetry; crosswinds; flight costs; groundspeed; migration; songbirds; tailwinds; wind support

Contents

1. Automated detection of vanishing bearings

2. Estimation of detection distance

3. Wind triangles

1. Automated detection of vanishing bearings

We measured the approximate vanishing bearings of birds departing on autumn migration from Kent Is. in 2009 and 2010 using a digital automated telemetry receiving array comprising three automated receiving stations located on the island (Fig. S1). Each station consisted of four 4-element Yagi antennas positioned at the top of a telescoping mast that stood 8 m high. All four antennas were connected to a single automated digital telemetry receiver (Model SRX-600, Lotek Wireless, Newmarket Ontario). Antennas on the southernmost receiving station were oriented towards 180°, 210°, 240°, and 270° relative to geographic north (Fig. S1). The middle and northern-most receiving stations were set-up in the same way but were shifted 10° and 20° towards the north, respectively. Given the position of the towers on the island this resulted in us scanning at orientations of 182°, 190°, 203°, 210°, 220°, 233°, 239°, 250°, 262°, 268°, 280°, and 291° relative to the middle tower and assuming a detection distance of 9.8 km (see *Estimate of detection range* and Fig. S3). In 2010 we rotated each tower 10° northward, based on the results of 2009 (Fig. S2). This resulted in us scanning at orientations of 191°, 200°, 213°, 220°, 230°, 243°, 249°, 260°, 271°, 278°, 280°, and 300° relative to the middle tower and again assuming a detection distance of 9.8 km.

Antennas were numbered from one to four, with one representing the southern-most facing antenna through to four, representing the northern most facing antenna (Fig. S1). Receivers were programmed so that antenna one would scan for a single radio frequency for 5.4 s, before switching to antenna two and scanning for the same frequency for 5.4 s and so on to antenna four. When the fourth antenna was done scanning, the cycle would repeat on the second and third frequencies before returning to the first. Receivers were synchronized so that antennas with the same number on each tower were scanning at the same time. This setup meant that all

three radio frequencies were scanned for a total of 21.6 s within every 64.8 s interval. Vanishing bearings were estimated using the last antenna to receive a signal from each departing bird before they moved out of range of the receivers.

2. Estimation of detection distance

We estimated the maximum horizontal distances out to which we detected birds as they departed Kent Is. in 2010, by multiplying a bird's groundspeed (m/s) by the amount of time we recorded a signal for a bird before it flew out of range of the receiving stations on the island (See [1]). We measured groundspeed by dividing the distance from the island to the estimated location that it arrived on the coast by the amount of time it took to reach the coast. This resulted in a median detection distance of 9.8 km with the 4-element Yagi antennas (Fig. S3). Given that doubling the number of elements on a Yagi antenna increases the range of the antenna by a factor of approximately 0.5, we estimate that the 9-element antennas associated with the automated receiving stations on the coast had detection ranges of approximately 15 km.

3. Wind triangles

Tail and crosswind components were calculated using a simple vector addition model (Fig. S4). Both measures simultaneously account for wind speed and direction. In these models, track direction and groundspeed represent the direction the bird is flying to reach a given destination relative to the origin and the speed at which they are flying over the ground, respectively. Wind direction and speed represent the direction the wind is blowing towards (not from) and the speed at which it is blowing relative to the ground, respectively. Heading direction represents the direction the bird has to face and fly in order to maintain its track direction relative to the wind.

Airspeed represents the bird's flight speed in the absence of any winds.

We derived tailwind components using the formula V_w*cos(β), where V_w is wind speed (m/s) and β is the difference between track and wind directions. Tailwind components can be either negative or positive and represent the amount that groundspeed along a given flight track is either reduced (headwinds) or increased (tailwinds), respectively, and has the units (m/s). A positive tailwind component is illustrated in Figure S4a, where the wind is blowing partly in the direction the bird is travelling. The net result of this positive tailwind component is an increase in groundspeed by the amount indicated by the hatched line along the bird's flight track. A negative tailwind component is illustrated in Figure S4b, where the wind is blowing partly in the direction from which the bird originated, resulting in a negative tailwind component and a reduction in groundspeed by an amount indicated by the hatched line associated with the bird's flight track. We derived crosswind components using the formula V_w*sin(β). The crosswind component also has the units m/s. With this measure, increasingly negative and positive crosswind components both represent increasingly strong side winds (winds blowing perpendicular to an individual's track direction); therefore, crosswind components always take on absolute positive values. The crosswind component in Fig. S4 is the same in both panels.

REFERENCES

1. Mitchell GW, Newman AE, Wikelski M, Norris DR. Timing of breeding carries over to influence migratory departure in a songbird: an automated radiotracking study. J Anim Ecol. 2012;81:1024-1033.

2. Dodge S, Bohrer G, Weinzierl R, Davidson SC, Kays R, Douglas D, Cruz S, Hans J, Brandes D, Wikelski M. The environmental-data automated track annotation (Env-DATA) system: linking animal tracks with environmental data. Movement Ecology. 2013;1(3)

Table S1. AICc model selection results for best fitting linear mixed effects model relating flight time across the ocean to tail and crosswind components from different altitudes. Wind components were derived from the NCEP/NOAA dataset (3 h, 32 km resolution; †) and were accessed through the Environmental-Data Automated Track Annotation Service provided by Movebank (www.movebank.org; [2]). Altitude refers to geo-potential height and standard deviation (SD) reflects day to day variation in altitude associated with the vertical movement of air pressure within the atmosphere. W represents the Akaike weights and Cumulative W represents cumulative Akaike weights. LL refers to log-likelihood. ^{2} indicates a curvilinear term was included in the model.

Model	Altitude ± SD (m)	K	AICc	ΔAICc	W	Cumulative W	LL
†tail	164.23 ± 43.82	4	245.11	0	0.49	0.49	-117.68
†tail	375.92 ± 44.09	4	245.88	0.77	0.33	0.83	-118.07
†tail	591.62 ± 44.58	4	248.51	3.4	0.09	0.92	-119.39
†tail + cross + cross^2	811.76 ± 45.32	6	249.29	4.18	0.06	0.98	-116.64
†tail x cross + tail^2 + cross^2	1503.67 ± 49.82	8	252.39	7.29	0.01	0.99	-114.41
†tail x cross	1036.74 ± 46.31	6	254.58	9.47	0	1	-119.29
†tail + cross + cross^2	1267.11 ± 47.78	6	254.82	9.71	0	1	-119.41
†tail x cross + tail^2	2256.10 ± 57.79	7	260.53	15.43	0	1	-120.47
†tail	1747.38 ± 52.41	4	263.32	18.22	0	1	-126.79
†tail	1997.96 ± 54.97	4	263.51	18.4	0	1	-126.88

†tail x cross	10	6	263.66	18.55	0	1	-123.83
†tail x cross	30	6	263.81	18.7	0	1	-123.9
Null	NA	3	268.05	22.94	0	1	-130.52

Table S2. AICc model selection results for best fitting linear model relating flight time along the coast to tail and crosswind components from different altitudes. Wind components were derived from the NCEP/NOAA dataset (3 h, 32 km resolution; †) and were accessed through the Environmental-Data Automated Track Annotation Service provided by Movebank (www.movebank.org; [2]). Altitude refers to geo-potential height and standard deviation (SD) reflects day to day variation in altitude associated with the vertical movement of air pressure within the atmosphere. W represents the Akaike weights and Cumulative W represents cumulative Akaike weights. LL refers to log-likelihood. ^{2} indicates a curvilinear term was included in the model.

Model	Altitude ± SD (m)	K	AICc	ΔAICc	W	Cumulative W	LL
†tail+tail^2	816.86 ± 45.70	4	171.83	0	0.65	0.65	-80.58
†tail+tail^2	1041.39 ± 46.55	4	174.09	2.26	0.21	0.86	-81.71
†tail+tail^2	597.11 ± 45.06	4	176.1	4.27	0.08	0.94	-82.72
†tail x cross	1507.43 ± 49.69	5	178.81	6.98	0.02	0.96	-82.26
†tail+tail^2	1271.32 ± 47.80	4	179.28	7.46	0.02	0.98	-84.31
†tail+tail^2	381.62 ± 44.52	4	180.71	8.88	0.01	0.98	-85.02
†tail+tail^2	170.65 ± 44.38	4	181.31	9.48	0.01	0.99	-85.32
†tail x cross	2000.88 ± 54.57	5	182.48	10.65	0	0.99	-84.1
†tail x cross	1750.50 ± 52.18	5	183.17	11.34	0	0.99	-84.44
†tail	10	3	183.2	11.37	0	1	-87.85

†tail x cross	2258.76 ± 57.19	5	183.3	11.47	0	1	-84.51
†tail + $tail^2$	30	3	184.16	12.33	0	1	-88.33
Null	NA	2	192.79	20.96	0	1	-94.04

Table S3. Results for models describing the effects of age and flight stage on the tailwind component experienced aloft. Models were fit with tailwind component measured at a pressure level of 1000mbar for the ocean flight and 925 mbar for the coastal flight, with the tailwind component measured from only 1000mbar for both flight stages, or with the tailwind component measured from 925 mbar for both flight stages. All wind data was from the NCEP/NOAA database and was accessed through the Environmental-Data Automated Track Annotation Service provided by Movebank (www.movebank.org; [2]).

Model	Parameter	β	SE	df	t	P
1000mbar + 925 mbar	Intercept	2.42	1.23	33.98	1.97	0.058
	Age	3.49	1.31	15.48	2.66	0.017
	Flight stage	-3.03	0.77	17.72	-3.96	0.001
1000 mbar only	Intercept	2.06	1.14	29.71	1.81	0.080
	Age	3.46	1.34	21.22	2.59	0.017
	Flight stage	-2.56	0.44	17.89	-5.74	<0.001
925 mbar only	Intercept	2.40	1.36	31.72	1.76	0.087
	Age	3.66	1.60	26.15	2.30	0.030
	Flight stage	-3.10	0.65	18.57	-4.79	<0.001

FIGURE CAPTIONS

Figure S1. Map of Kent Is. depicting the locations and orientation of antennas on each station from 2009. Orientation of the antennas is relative to geographic north. The orientation of the southern-most receiving station is 180° and the western most antenna is oriented towards 270°. The two middle antennas are separated from the other two antennas and each other by 30° and are oriented towards 210° and 240°. The antenna at the middle and northern most station are shifted 10° and 20°, respectively, from the southern-most station. Antennas were numbered one through four. All three stations were synchronized such that antenna one at each of the stations was scanning at the same time, then antenna 2, 3, and 4, at which point, the cycle would repeat on the second VHF radio frequency, and then the third.

Figure S2. Rose diagrams (circular histograms) illustrating vanishing bearings of Savannah sparrows departing Kent Is. in (a) 2009 and (b) 2010. Vanishing bearing were measured and are illustrated in approximately 10 degree intervals and are based on the orientation of the last antenna to receive a signal from each bird before they flew out of range of the automated receivers on Kent Is. The labels along the 90° axis indicate the number of birds represented by each concentric circle. The solid black line and arrow represents the median direction of departure and the length of the arrow represents the amount of circular dispersion (rho) multiplied by 15 (i.e., the maximum number of observations observed for an individual bin in each year).

Figure S3. Density plot of estimated detection distances for Savannah sparrows departing Kent Is. in 2010. Estimates were as calculated by multiplying the amount of time each bird was tracked once it departed Kent Is. by its groundspeed over the ocean. The hatched black line represents the median estimated detection distance (9.8 km).

Figure S4. Hypothetical wind triangles or vector addition diagrams illustrating (a) a positive tailwind component and (b) a negative tailwind component. Crosswind components are identical in both (a) and (b). Origin refers to where a bird started and destination refers where it is trying to get to. Track direction refers to a bird's trajectory from a place of origin relative to another reference point on the surface of the earth (destination). Groundspeed represents the speed at which the bird is flying over the ground. Wind direction and speed represent the direction the wind is blowing towards and its speed, respectively. Heading represents the direction in which a bird must fly to maintain a given track direction given wind speed and direction. Airspeed is the bird's flight speed in the absence of any winds. Speeds were measured in m/s. β refers to the difference between an individual's track direction and the wind direction (°). α refers to the difference between an individual's track and heading direction.

Figure S1.

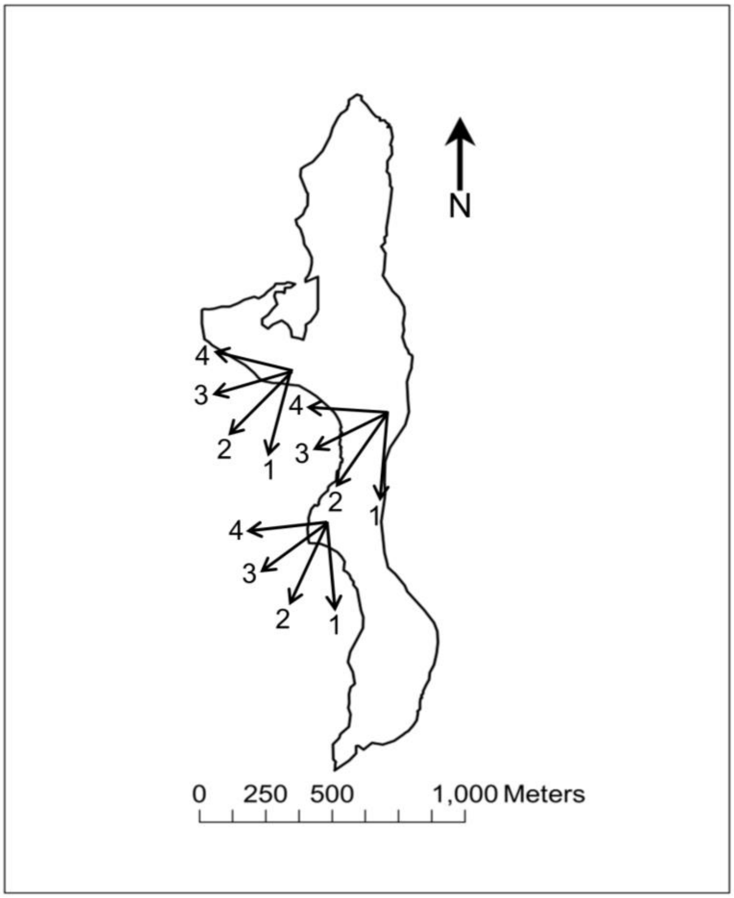

Figure S2.

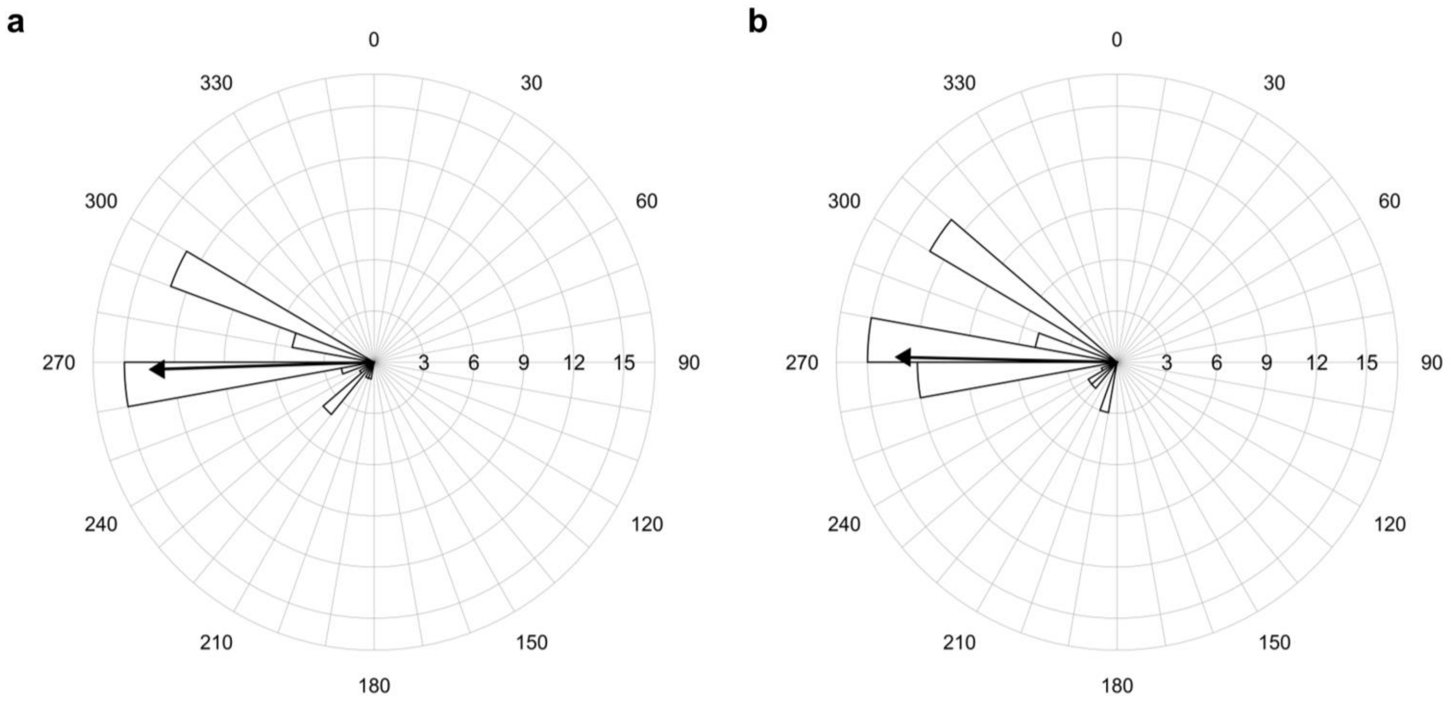

Figure S3.

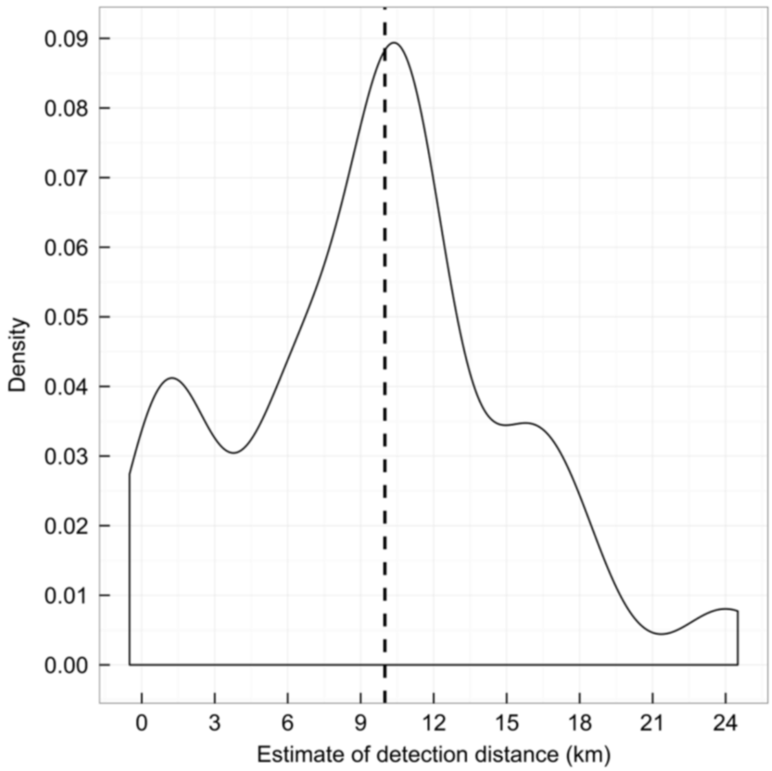
0.09
0.08
0.07
0.06
0.05
0.04
0.03
0.02
0.01
0.00
Density
0
3
6
9
12
15
18
21
24
Estimate of detection distance (km)

165 **Figure S4.**

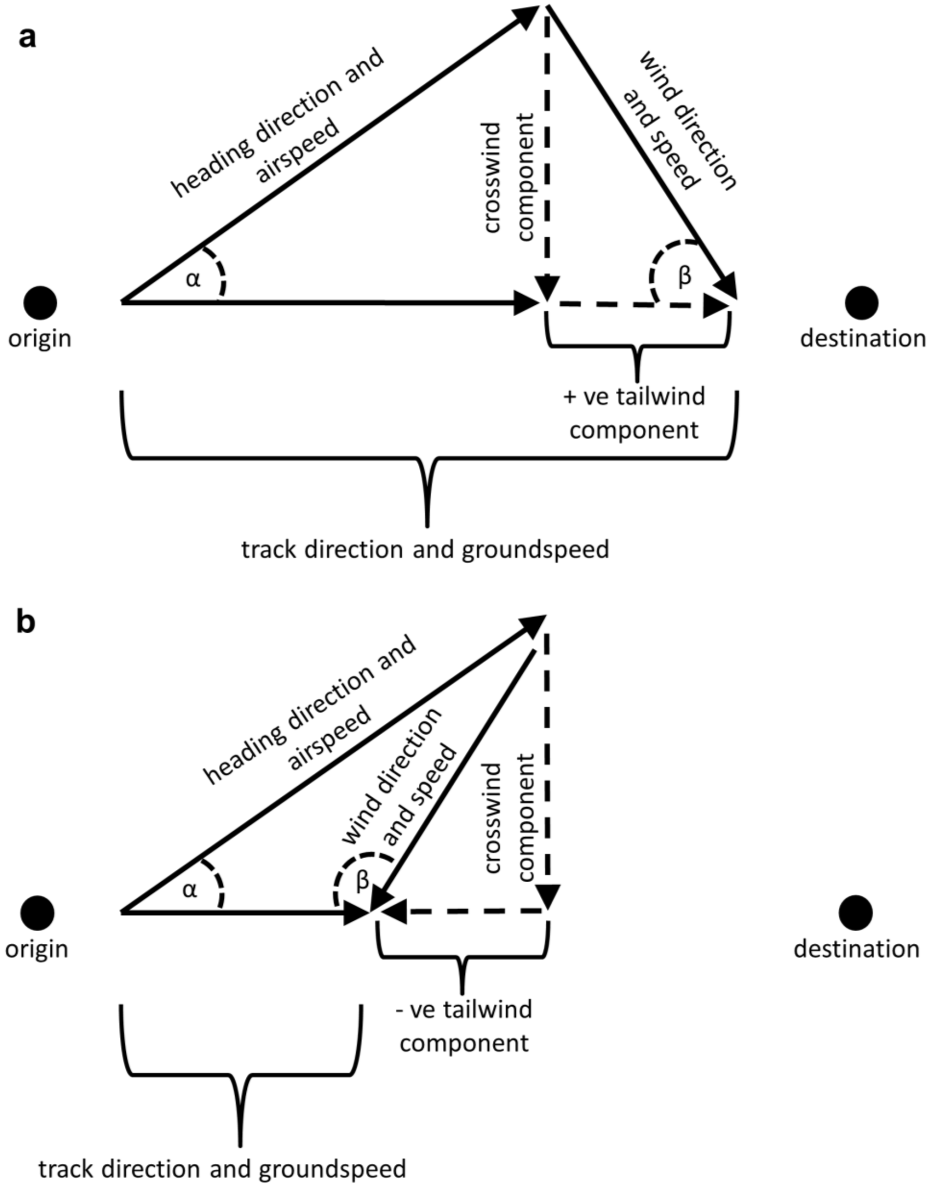

Supplement: Additional file 1: — Supporting information regarding automated detection of vanishing bearings, estimation of detection distance, and wind triangles. This file also contains the following tables and figures: Table S1. AICc model selection results for best fitting linear mixed effects model relating flight time across the ocean to tail and crosswind components from different altitudes; Table S2. AICc model selection results for best fitting linear model relating flight time along the coast to tail and crosswind components from different altitudes; Table S3. Results for models describing the effects of age and flight stage on the tailwind component experienced aloft; Figure S1. Map of Kent Is. depicting the locations and orientation of antennas on each station from 2009; Figure S2. Rose diagrams (circular histograms) illustrating vanishing bearings of Savannah sparrows departing Kent Is. in 2009 and 2010; Figure S3. Density plot of estimated detection distances for Savannah sparrows departing Kent Is. in 2010; Figure S4. Hypothetical wind triangles or vector addition diagrams illustrating a (A) positive and (B) negative tailwind component. [file 40462_2015_46_MOESM1_ESM.pdf]
